# Maternal diet quality and nutrient intakes across preconception and pregnancy are not consistent with Australian guidelines: Results from the pilot BABY1000 study

**DOI:** 10.1002/fsn3.3401

**Published:** 2023-05-04

**Authors:** Katie Maneschi, Taryn Geller, Clare E. Collins, Adrienne Gordon, Allison Grech

**Affiliations:** ^1^ School of Life and Environmental Sciences, Faculty of Science University of Sydney Camperdown New South Wales Australia; ^2^ School of Health Sciences, Faculty of Health and Medicine University of Newcastle Callaghan New South Wales Australia; ^3^ Priority Research Centre for Physical Activity and Nutrition University of Newcastle Callaghan New South Wales Australia; ^4^ Central Clinical School, Faculty of Medicine and Health University of Sydney Camperdown New South Wales Australia; ^5^ Charles Perkins Centre University of Sydney Camperdown New South Wales Australia

**Keywords:** diet quality, maternal nutrition, micronutrients, preconception, pregnancy

## Abstract

Background and objective: Maternal nutrition has profound and lasting effects on growth and health from infancy into adulthood. The aim of this manuscript was to assess diet quality and nutrient adequacy in preconception and pregnancy in BABY1000 pilot study participants (*n* = 171). Study design and methods: The Australian Eating Survey (AES) Food Frequency Questionnaire was administered to women based in Sydney, Australia, at preconception or 12 weeks' gestation (*n* = 158), and again at 36 weeks' gestation (*n* = 99). Primary outcomes were diet quality and nutrient intake. Diet quality was evaluated using the AES diet quality subscale, the Australian Recommended Food Score (ARFS). Nutrient intakes were compared to Australian Nutrient Reference Values. Diet quality and nutrient intakes were not consistent with Australian recommendations. Over 83% of women exceeded the suggested target limits for percentage energy from saturated fat. Median ARFS was 37 at baseline, and 38 in late pregnancy (maximum score 73). Inadequate micronutrient intakes from food were common; no participants met the Estimated Average Requirement for iron, 76%–84% for iodine, 70%–78% for calcium and 44%–50% for folate. Maternal diet quality and nutrient intakes in the current sample are inconsistent with pregnancy recommendations and therefore may not be supporting optimal perinatal or long‐term offspring health. Stronger messaging around the importance of prenatal nutrition, prevalence of dietary inadequacy, and availability of reliable support and information specific to nutrition in pregnancy is crucial in supporting women to improve their nutrition both before and during pregnancy.

## Graphical abstract

The aim was to assess diet quality and nutrient adequacy across preconception and pregnancy in women from the BABY1000 pilot study (*n* = 171) using the Australian Eating Survey (AES) Food Frequency Questionnaire. Diet quality and nutrient intakes were not consistent with Australian recommendations; 99%–100% of women did not meet the Estimated Average Requirement for iron, 76%–84% for iodine, 70%–78% for calcium, and 44%–50% for folate. Improvements in prenatal care and public health policy supporting women's nutrition are required to optimize maternal diet and nutrition‐related health outcomes in pregnancy.

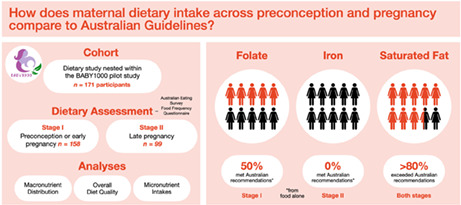

## 1 INTRODUCTION

From the earliest stages of conception, maternal nutrition influences fetal growth and long‐term offspring health (Barker, 1995; Stephenson et al., 2018). The importance of adequate micronutrient supply at critical windows of fetal development is well‐established, especially regarding folate before and during early pregnancy for prevention of neural tube defects (NTDs) (Berti et al., 2011; Mousa et al., 2019). The effects of poor fetal nutrition persist beyond infancy through epigenetic modifications that increase offspring susceptibility to later life chronic diseases, including type 2 diabetes and cardiovascular disease (Gluckman et al., 2009; Painter et al., 2008). Maternal nutrition before and during pregnancy plays a key role in the perinatal and long‐term health of future Australians.

Poor adherence to Australian nutritional recommendations is consistently reported (Beringer et al., 2021; Blumfield et al., 2011; Hure et al., 2009; Lee, Collins, et al., 2018). Overrepresentation of saturated fat and energy‐dense, nutrient‐poor foods has been observed in a small sample of pregnant Indigenous Australians from the *Gomeroi gaaynggal* cohort (*n* = 58) in 2018 (Lee, Collins, et al., 2018), as well as in a large nationally representative sample of preconceptional (*n* = 454) and pregnant (*n* = 606) women from the Australian Longitudinal Study on Women's Health (ALSWH) in 2009 (Blumfield et al., 2011; Hure et al., 2009). Suboptimal intakes of iron, calcium, and folate were also reported in both samples (Blumfield et al., 2011; Hure et al., 2009; Lee, Collins, et al., 2018; Slater et al., 2020), as well more recently, in a cohort of women recruited as part of the *Gomeroi gaaynggal* project (*n* = 152) (Beringer et al., 2021).

The temporal effects of early‐life exposures on future offspring health outcomes remain understudied. The BABY1000 pilot study hopes to better understand these effects by collecting data and biological samples that pertain to diet and other health indicators from preconception through pregnancy and early life. Understanding patterns in maternal diet quality and nutrient intakes across preconception and pregnancy in a prospective cohort with collection of birth and infant health outcomes may help to clarify the relationship between maternal nutrition and offspring health and inform future interventional studies. Future publications hope to explore the impact of maternal nutrition on other health outcomes, such as infant growth and the developing gut microbiome. In the current sample of preconception and pregnant women from the BABY1000 pilot, the study aims were to assess: (i) maternal diet quality; (ii) macronutrient intakes; and (iii) adequacy of usual food‐derived micronutrient intakes.

## 2 METHODS

### 2.1 Data collection

This dietary study was nested within the BABY1000 pilot study, a prospective longitudinal cohort of mother–infant pairs observed from preconception through pregnancy and early life. Biological samples, clinical measurements, and questionnaires that pertain to maternal and/or paternal diet, lifestyle, mental well‐being, health literacy, and infant health are collected before pregnancy up until the infant's second birthday. Women were recruited during preconception or the first trimester of pregnancy from Royal Prince Alfred (RPA) Hospital and the Charles Perkins Centre RPA antenatal clinic in Sydney, Australia, and written informed consent was provided. The study is ongoing at the time of publication and aims to pilot a prospective longitudinal cohort study (including core collection of biological samples) assessing maternal nutrition and impact on infant health up to 2 years of age. The pilot will assess recruitment, feasibility, and acceptability of enrolment in an early pregnancy cohort and the methodology, collection and storage of study data and biological samples (online questionnaires via tablets, body composition, blood, saliva, buccal swabs and stool). The information gained from the pilot will inform larger studies and collaboration with other Australian cohort studies (Gordon, 2017). Ethics approval for the study was obtained from Sydney Local Health District (RPAH zone) Review Committee: Protocol no. X170019.

### 2.2 Australian eating survey food frequency questionnaire

Maternal dietary intake was assessed during preconception and/or pregnancy using the Australian Eating Survey (AES) Food Frequency Questionnaire (FFQ) (Collins et al., 2014). This self‐administered, semiquantitative FFQ is designed to assess usual dietary intake over the previous 3–6 month period and has been validated among Australian adults (Burrows et al., 2015; Collins et al., 2014). The first dietary assessment was administered at recruitment (preconception or at 12 weeks' gestation), designed to reflect habitual prepregnancy dietary patterns. This assessment point will be referred to as “preconception/early pregnancy” due to overlap in dietary assessment windows at these time points. A second dietary assessment was completed at 36 weeks' gestation (“late pregnancy”) to represent usual intake during pregnancy and is aligned with the final study visit during pregnancy.

The AES FFQ examines usual intake of 120 commonly consumed foods and beverages over the previous 3–6‐month period, with frequency options ranging from “never” to “more than 7 times per day” (Collins et al., 2014). The AUSNUT 2011–13 database was used to compute nutrient intakes from the AES FFQ output (Food Standards Australia New Zealand: AUSNUT 2011‐13 Food Nutrient Database, 2016). Supplementary questions pertain to age, food behaviors, activity level, and use of dietary vitamin and/or mineral supplements (Collins et al., 2014), although these dietary supplement questions are not specific to brand or micronutrient content or dose and hence cannot be used to calculated nutrient intake with accuracy. Further details of the AES FFQ are provided elsewhere (Collins et al., 2014).

### 2.3 Australian recommended food score

The Australian Recommended Food Score (ARFS) is a food‐based diet quality index derived using data from the AES FFQ. The Australian Recommended Food Score awards points on the basis of the variety within nutrient‐dense “core” foods outlined in the Australian Guide to Healthy Eating (AGHE) (Ashton et al., 2017; Collins et al., 2015). The ARFS is scored out of 73 points and provides a proxy measure for overall diet quality, with higher score in young adults related to higher skin and plasma concentrations of dietary carotenoid found in a range of vegetables and fruit (Ashton et al., 2017; Collins et al., 2015). The component scores for food groups within ARFS indicate relative adherence to AGHE recommendations regarding core food groups. Higher scores indicate higher diet quality and alignment with AGHE recommendations for adults (Collins et al., 2015). A score above 47 points is classified as “outstanding”; a score of 39–46 points is “excellent”; a score of 33–38 is “getting there”; and a score below 33 “need[s] work” (Williams et al., 2017).

### 2.4 Sample

The current findings report on the pooled FFQ responses available from the pilot cohort sample recruited up to December 2020. A total of 274 AES FFQ entries were accessed from *n* = 171 after deleting biologically implausible responses. Survey responses with biologically plausible estimates of total energy intake (4.5–20 MJ/day) (Meltzer et al., 2008) were included in the analyses (*n* = 257). Usual energy intakes outside these values are suggestive of a major degree of over‐ or underreporting and were excluded (*n* = 17) (Meltzer et al., 2008). The present sample consists of a total of 171 women, 86 of whom had provided dietary data during both preconception/early pregnancy and late pregnancy. Demographic data pertaining to age, gravidity, marital status, education, employment, and smoking were collected from women at study entry.

### 2.5 Australian guide to healthy eating

The AGHE is Australia's national food selection guide that provides recommendations for daily consumption of foods belonging to the “core” food groups, including: grains (i.e., breads and cereals), vegetables (including legumes), fruit, dairy (and alternatives), and meat (including vegetarian alternatives) (NHMRC, 2006). The AGHE also provides a suggested consumption limit for “non‐core” or energy‐dense, nutrient‐poor food items, such as chocolate and ice‐cream (NHMRC, 2006). For pregnancy, there is a suggested limit of 2.5 serves/day or ~1500 kJ/day for these foods, based on the understanding that 1 serve = 600 kJ (NHMRC, 2006). The AGHE serving recommendations are stratified by age, gender, and life‐stage.

### 2.6 Nutrient reference values

The Nutrient Reference Values (NRVs) for Australia and New Zealand prescribe gender‐ and life stage‐specific nutrient intake targets for optimal health and prevention of nutrient deficiency (NHMRC, 2006). The Estimated Average Requirement (EAR) is the level of intake expected to meet the requirements of half the healthy individuals within a population subgroup (NHMRC, 2006). The EAR is the most suitable NRV for estimating proportions of adequacy in population intakes (NHMRC, 2006). The Acceptable Macronutrient Distribution Range (AMDR) is the ideal intake range of a certain macronutrient that is estimated to facilitate adequate intake of the remaining macronutrients and promote health (NHMRC, 2006).

### 2.7 Data analysis

Primary outcome measures included overall diet quality, macronutrient distribution, and micronutrient intakes, examined in preconception/early pregnancy and again in late pregnancy. Diet quality was described by the total ARFS and component scores, as well as percent energy contribution of core and noncore food items. Macronutrient intake, expressed as a percentage of total energy intake, was compared to the AMDR (NHMRC, 2006). Six micronutrients with substantial perinatal importance were selected for analysis (vitamin B12, calcium, folate, iodine, iron and zinc) (Blumfield et al., 2013). Micronutrient intake from food sources only was assessed relative to the EAR. Where dietary supplement data were available, trends in usage were described across preconception and early pregnancy (*n* = 158), and late pregnancy (*n* = 99).

All statistical analyses were performed using IBM SPSS Statistics, Version 20.01.0.14 (SPSS for Windows, IBM Corp). Normality within each nutrient intake category was tested using Kolmogorov–Smirnov and Shapiro–Wilk tests, with significance >0.05 indicating normal data spread. As not all variables were normally distributed, the median and Interquartile Range (IQR) were reported for consistency. The Wilcoxon signed‐ranks test was applied for comparisons in nutrient intake across pregnancy, with *α* ≤ 0.05. Micronutrient intake from food alone was also assessed relative to the ARFS at late pregnancy (*n* = 99). Women were stratified into tertiles based on total ARFS and a comparison was drawn between women within the lowest ARFS tertile (Tertile 1, *n* = 37) and those within the highest ARFS tertile (Tertile 3, *n* = 32) using two‐sample t‐tests assuming unequal variances. Demographic variables (age, gravidity, country of birth, education, and employment status) and Body Mass Index (BMI) are presented descriptively (number and percentage) and were assessed in relation to diet quality (ARFS) at 12 weeks and 36 weeks using multivariate logistic regression. ARFS categories were dichotomized according to the categories defined by the AES: “outstanding/excellent” (score of 39 or above) and “getting there/needs work” (score of 38 or below). Characteristics were also dichotomized, as follows: born in Australia vs. overseas; aged <35 years vs. >35 years; pregnant before (multiparous) vs. first pregnancy (primiparous); employed in paid work vs. not employed in paid work; education at tertiary level or higher vs. education below tertiary level; Body Mass Index ≤24.99 kg/m^2^ vs. ≥25 kg/m^2^. Results are reported as adjusted odds ratios (aOR) with 95% confidence intervals (CI), and significance set at *p* < 0.05.

## 3 RESULTS

Full demographic characteristics of women included in the current analyses are summarized in Table 1. The median age of women in the pilot BABY1000 study was 33 years. There were similar numbers of primigravid (52%, *n* = 66/128) and multigravid (48%, *n* = 62/128) women. Most women had attained tertiary level education (96%, *n* = 129/134). Of those with anthropometric data available (*n* = 90/162), 56% had a BMI in the “healthy” range (18.5–24.9 kg/m^2^).

**TABLE 1:** Demographic characteristics of women from the pilot BABY1000 study with AES data available (*n* = 171).

Characteristic	*n*	%
Age^a^ (years)	33 (20–54; 5)	
Gravidity		
Primigravid	89	54
Multigravid	77	46
Missing	5	–
Country of birth		
Australia	86	50
Overseas	85	50
Ethnicity		
European	120	56
South Asian	26	12
East Asian	26	12
South East Asian	17	8
Middle Eastern	5	2
African	1	0.5
Pacific Islander	4	2
Other	14	7
Missing	12	–
Marital status		
Married	102	74
Defacto	32	23
Other (not living with partner)	2	1.5
Single	1	0.7
Missing	34	–
Education		
Beyond high school	129	96
High school or lower	5	4
Missing	37	–
Employment		
Current employed	138	93
Not currently employed	10	7
Missing	4	–
BMI		
≤18.5 kg/m^2^	5	3
18.5–24.9 kg/m^2^	90	56
25–29.9 kg/m^2^	50	31
≥30 kg/m^2^	17	10
Missing	9	‐
Smoking		
Current smoker	0	0
Non‐smoker	130	100
Missing	41	–

Abbreviations: BMI, body mass index; IQR, interquartile range.

^a^ Age reported as median (range; IQR); −, Percent is not calculated for missing values.

### 3.1 Dietary intake and diet quality

Results from the AES FFQ showed mean macronutrient distributions of 18% protein, 45% carbohydrate, and 36% total fat, with 13% from saturated fat (Table 2) in preconception/early pregnancy. In late pregnancy, mean macronutrient distributions of 17% protein, 45% carbohydrate, and 37% total fat, with 14% from saturated fat, were observed. Core foods made up 72% of total energy intake in preconception/early pregnancy and in late pregnancy (Table 2), while noncore foods contributed 28%.

**TABLE 2:** Diet quality scores and nutrient intake relative to the Australian NRVs in women from the pilot BABY1000 study (*n* = 171).

	Preconception/early pregnancy (*n* = 158)	Late pregnancy (*n* = 99)
	Median (IQR)	Median (IQR)
Total energy (kJ)	8338 (6831–10,106)	8438 (7421–10,276)
Core foods (kJ)	5953 (4969–6939)	6209 (4836–7234)
Core foods (% energy)	72 (65–78)	72 (64–78)
Noncore foods (kJ)	2345 (1662–3049)	2355 (1787–3366)
Noncore foods (% energy)	28 (22–35)	28 (22–36)
ARFS (max. points awarded)		
Total (73)	37 (32–42)	38 (34–44)
Vegetables (21)	14 (11–17)	14 (11–17)
Grains (13)	6 (5–7)	6 (5–8)
Fruit (12)	7 (5–8)	7 (5–8)
Dairy (11)	3 (3–5)	5 (3–6)
Meat (7)	3 (2–3)	3 (2–4)
Meat alternatives (6)	3 (2–3)	3 (2–4)
Extras (2)	1 (0–1)	1 (0–1)
Water (1)	1 (1–1)	1 (1–1)

Micronutrient	EAR	Median (IQR)	Meeting *n* (%)	Below *n* (%)	Median (IQR)	Meeting *n* (%)	Below *n* (%)
Vitamin B12 (μg)	2.2	3.4 (2.5–4.5)	125 (79)	33 (21)	3.6 (2.9–4.5)	85 (86)	14 (14)
Calcium (mg)	1050	799 (553–1013)	34 (22)	124 (78)	838 (690–1094)	30 (30)	69 (70)
Folate (μg)^a^	520	521 (389–656)	79 (50)	79 (50)	541 (411–655)	55 (56)	44 (44)
Iodine (μg)	160	119 (87–149)	26 (16)	132 (84)	130 (96–157)	24 (24)	75 (76)
Iron (mg)	22	10 (8–13)	2 (1)	156 (99)	11 (9–13)	0 (0)	99 (100)
Zinc (mg)	9	10 (8–13)	118 (75)	40 (25)	11 (9–13)	83 (84)	16 (16)

Macronutrient	AMDR	Median^b^ (IQR)	Within *n* (%)	Above *n* (%)	Below *n* (%)	Median^b^ (IQR)	Within *n* (%)	Above *n* (%)	Below *n* (%)
Protein	15–20	18 (15–20)	128 (81)	3 (2)	27 (17)	17 (16–19)	87 (88)	1 (2)	11 (11)
Carbohydrate	45–65	45 (41–50)	84 (53)	1 (1)	73 (46)	45 (41–50)	53 (54)	0 (0)	46 (46)
Total fat	20–35	36 (33–39)	73 (46)	84 (53)	1 (1)	37 (33–40)	35 (35)	64 (65)	0 (0)
Saturated fat	≤10	13 (12–15)	26 (16)	132 (83)		14 (12–16)	14 (14)	85 (86)	

Abbreviations: AMDR, Acceptable Macronutrient Distribution range; ARFS, Australian Recommended Food Score; EAR, Estimated Average Requirement for pregnant women aged 19–50 years; IQR, Interquartile Range; NRVs, Nutrient Reference Values.

^a^ Dietary folate equivalents.

^b^ Macronutrient intake expressed as a percentage of total energy intake.

The median ARFS was 37 in preconception/early pregnancy, and 38 in late pregnancy (Table 2). These values correspond to an overall classification for diet quality as “getting there.” Relative to the total points available for each component, vegetables scored most highly (14 out of 21 points), followed by fruit (7 out of 12 points). The remaining food groups (grains, dairy, meat, and meat alternatives) scored less than half the total points available across preconception/early pregnancy and late pregnancy. Dairy foods were scored most poorly in this sample, with median scores of 3 and 5 points (out of 11) in preconception/early pregnancy and late pregnancy, respectively.

Across pregnancy, there was a significant increase in percentage energy contribution from saturated fat (*p* < 0.01) in those with paired data available (Tables S1 and S2). No significant differences were identified in energy contribution from remaining macronutrients (carbohydrate, protein, and fat) across pregnancy (Tables S1 and S2). No significant differences in micronutrient intake were observed across pregnancy with exception to iodine intake, which increased significantly across pregnancy (*p* < 0.002) (Tables S1 and S2). Core foods had a signficiantly lower contribution to total energy intake across pregnancy, while there was significantly greater contribution from noncore foods (*p* < 0.024) (Tables S1 and S2).

Across preconception and pregnancy, median dietary intakes were above the EAR for vitamin B12, folate and zinc, and below the EAR for calcium, iodine and iron (Table 2). No women achieved the EAR for all six micronutrients studied (data not shown). Adequate levels of vitamin B12 and zinc were acquired through diet in more than 75% of women (Table 2). Inadequate micronutrient intakes from food were common across preconception/early pregnancy and late pregnancy, with 99%–100% of women not meeting the EAR for iron, 76%–84% for iodine, 70%–78% for calcium, and 44%–50% for folate. Median iron intake was less than half the EAR within both timeframes, and only one woman had an intake above the EAR. Most women used at least one multivitamin or single nutrient supplement in preconception/early pregnancy (94%) and late pregnancy (91%) (Table 3). Supplements containing folic acid were used most frequently in preconception/early pregnancy (91%), followed by iodine‐containing supplements (86%) (Table 3). Iron‐containing supplements were the most commonly used supplement in late pregnancy (86%) (Table 3).

**TABLE 3:** Dietary supplement use in women from the pilot BABY1000 study in preconception/early pregnancy (*n* = 158) and late pregnancy (*n* = 99).

Supplement 1	Preconception/early pregnancy (*n* = 158)		Late pregnancy (*n* = 99)	
	*n*	%	*n*	%
Not supplementing	8	6	7	9
Supplementing 2	117	94	70	91
Missing	33	–	22	–
Supplement containing				
Vitamin B12	98	78	54	70
Calcium	95	76	55	71
Folic acid	114	91	61	79
Iodine	107	86	55	71
Iron	99	79	66	86
Zinc	95	76	52	68

*Note*: 1 Values are not mutually exclusive due to the usage of multiple supplements; 2, Use of one or more multivitamin or single nutrient supplements; −, Percent is not calculated for missing values.

Significantly greater intakes of all micronutrients examined from food alone were observed in late pregnancy in tertile 3 (women with higher ARFS, >42) relative to tertile 1 (women with lower ARFS, <36), except for zinc (Tables S2).

### 3.2 Comparison of diet quality by demographic characteristics and BMI

Multivariate logistic regression results found that participants born in Australia were significantly more likely to a higher ARFS (“outstanding/excellent”) at baseline (preconception or 12 weeks' gestation) compared to those born overseas (OR 0.47, 95% CI 0.24–0.94, *p* = 0.03). At 36 weeks' gestation, multiparity was associated with a higher diet quality (OR 0.39, 95% CI 0.16–0.96, *p* = 0.04). No other variables in the model were significantly associated with diet quality, as measured by the ARFS, at either 12 weeks or 36 weeks (Table 4).

**TABLE 4:** Multivariate logistic regression of Australian Recommended Food Score (ARFS) category (“outstanding/excellent” vs. “getting there/needs work”) by demographic characteristics at preconception or 12 weeks' gestation (*n* = 148) and 36 weeks' gestation (*n* = 94) in BABY1000 participants.

	ARFS at preconception or 12 weeks (*n* = 148)			ARFS at 36 weeks (*n* = 94)		
Characteristic	*n* (%)	aOR	95% CI	*n* (%)	aOR	95% CI
Born in Australia	80 (54%)	0.47*	0.24–0.94	52 (55%)	0.63	0.27–1.5
Pregnant before	73 (49%)	0.69	0.36–1.30	43 (46%)	0.39 *	0.16–0.96
Age 35 years or more^a^	56 (38%)	1.15	0.55–2.4	34 (36%)	1.8	0.71–4.4
Education at tertiary level or above	118 (80%)^a^	0.96	0.79–1.15	69 (73%)^a^	Not included in multivariate analysis – cell sizes <1	
Employed	117 (79%)^b^	0.95	0.79–1.15	69 (73%)^b^	Not included in multivariate analysis – cell sizes <1	
Body Mass Index ≥ 25 kg/m^2^	61 (41%)	1.15	0.57–2.31	37 (39%)	1.5	0.60–3.6

* *p*‐Value < 0.05.

^a^ Education data missing for *n* = 35 participants at preconception or 12 weeks and *n* = 28 participants at 36 weeks;

^b^ Employment data missing for *n* = 34 participants at preconception or 12 weeks and *n* = 27 participants at 36 weeks.

## 4 DISCUSSION

### 4.1 Macronutrient distribution and diet quality

The current findings indicate that the macronutrient distribution of usual diet is relatively high in total fat and low in carbohydrate across both preconception and pregnancy. Most women (>53%) exceeded the upper limit of the AMDR for total fat, while almost half (46%) reported carbohydrate intake below the lower limit of the AMDR. Percent energy from saturated fat was high (13%–14%), with the majority of women (>83%) exceeding recommendation to limit saturated fat to 10% or less of total energy intake (NHMRC, 2006).

The AGHE provides a recommended consumption limit for noncore food items (NHMRC, 2006). In pregnancy, there is a suggested limit of 2.5 serves/day or ~1500 kJ/day for these foods, based on the understanding that 1 serve = 600 kJ (NHMRC, 2006). In our sample, percent energy from these noncore foods was relatively high, contributing 28% of total energy intake in both preconception/early pregnancy and late pregnancy (Table 2), equivalent to ~2350 kJ. While energy requirements vary among women according to several variables (e.g., age, body composition, and genetic factors), our results indicate that 82% of women exceeded the suggested energy intake limit for these foods (~1500 kJ/day).

Blumfield et al. reported similar findings in a systematic review of macronutrient intakes across pregnancy in Australia, New Zealand, USA, Canada, UK, Europe, and Japan (Blumfield et al., 2012). Similarly, in a recent sample of women (*n* = 534) in their third trimester of pregnancy attending the antenatal outpatient clinics at John Hunter Hospital, Newcastle, New South Wales (NSW), only 7.3% of women achieved less than 10% daily energy from saturated fat (Slater et al., 2020). Our findings are attributed to higher‐than‐recommended intake of noncore foods, resulting in displacement of nutrient‐dense core foods from the diets of women within the BABY1000 cohort. High saturated fat consumption and inadequate intake of nutrient‐rich core foods may increase the propensity for possible adverse effects on mother‐infant cardiometabolic health (Williams et al., 2014). As healthy dietary habits formed prepregnancy are more likely to extend into future pregnancies, these findings emphasize the need for stronger nation‐wide initiatives to improve diet quality among women of reproductive age (Barker et al., 2018).

### 4.2 Micronutrient intake

**FIGURE 1:**
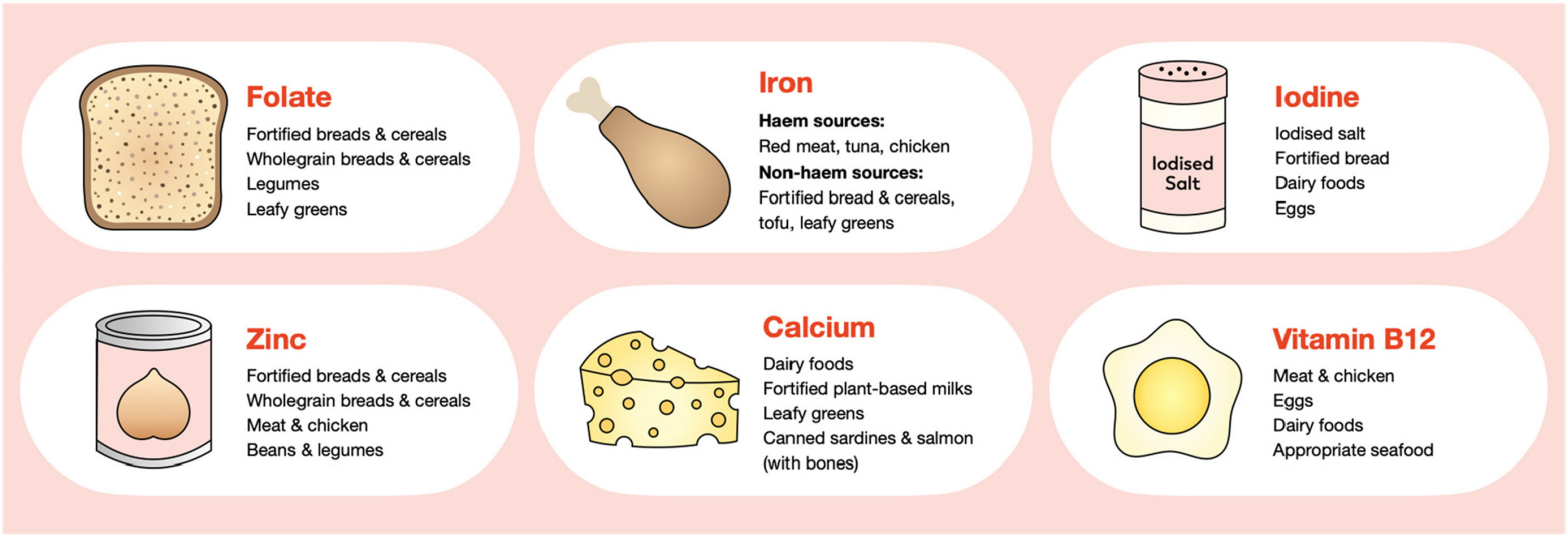
Micronutrients with substantial perinatal importance and their common food sources (Blumfield et al., 2013; Hermoso et al., 2011).

Micronutrients with importance in the perinatal period and their food sources are summarized graphically in Figure 1. No woman in this sample achieved the EAR for all micronutrients examined through food alone. No significant differences in micronutrient intake were observed across pregnancy with exception to iodine intake. Dietary intakes were commonly inadequate across preconception/early pregnancy and late pregnancy, with 99%–100% of women not meeting the EAR for iron, 76%–84% for iodine, 70%–78% for calcium, and 44%–50% for folate. Similar findings were observed in pregnant Indigenous Australians from the *Gomeroi gaaynggal* cohort (*n* = 58), with 98% of women failing to meet the EAR for iron, 47% for calcium, and 31% for folate (Lee, Collins, et al., 2018). In the sample of women (*n* = 534) from John Hunter Hospital, Newcastle, NSW, only four women met the NRVs for folate, iron, calcium, zinc, and fiber from food intakes alone (Slater et al., 2020). In a systematic review of micronutrient intakes across pregnancy in developed nations, Blumfield et al. reported suboptimal intakes of iron, folate, and calcium relative to country‐specific recommendations (Blumfield et al., 2013). Consistent findings were reported by Caut et al. in a recent review of micronutrient intakes across preconception and pregnancy in Australia, Canada, China, Europe, India, Japan, and Pakistan (Caut et al., 2020). Improving maternal nutrition during pregnancy should be a global priority to assist in mitigating the growing international burden of chronic disease (Balbus et al., 2013; Mendez & Kogevinas, 2011).

Examination of micronutrient intake from food alone in late pregnancy revealed that intakes of all micronutrients of interest (except zinc) were significantly higher in women within the highest ARFS tertile (ARFS > 2) relative to those in the lowest ARFS tertile (ARFS < 36). This is consistent with previous validation studies of the AES, which have shown correlation between ARFS and micronutrient intake (Blumfield et al., 2011; Slater et al., 2020).

#### 4.2.1 Calcium

With dairy being a primary source of dietary calcium, the suboptimal intakes of calcium in the present sample is largely attributed to low ARFS component scores for dairy foods across preconception/early pregnancy (4 out of 11 points) and late pregnancy (5 out of 11 points). The poor intake of calcium‐rich dairy foods in this sample is a concern, as adequate dietary calcium is associated with a reduced risk of pre‐eclampsia and preterm births, as well as improved maternal and infant bone health (Berti et al., 2011; Mousa et al., 2019).

#### 4.2.2 Iron

Iron deficiency is a global health concern, estimated to affect up to 52% of pregnant women worldwide (Abu‐Ouf & Jan, 2015). The third trimester of pregnancy brings elevated risk of iron deficiency, as usual metabolic demands are compounded by fetal iron deposition (Hermoso et al., 2011). Adequate dietary intakes of iron are important for the prevention of maternal iron deficiency anemia (IDA) and associated complications for the offspring and mother, including poor gestational weight gain, preterm birth, and maternal depression (Abu‐Ouf & Jan, 2015). Between March 2007 and January 2009, 18% of women (*n* = 461) within a cohort of 2654 from Northern Tasmania, Australia, were shown to have a moderate IDA (Khalafallah et al., 2010). In a large cohort of Australian women (*n* = 2146), Livock et al. found that total intake of iron, including diet and supplement use, was below the Recommended Daily Intake (RDI) in 68%–82% of women during preconception and pregnancy (Livock et al., 2017). Although the RDI may overestimate proportions of population inadequacy (NHMRC, 2006), in light of this finding, the dietary deficit in 99% of women in the present sample is a cause for concern despite seemingly satisfactory rates of iron supplementation (79%–86%). This dietary deficit may be attributed to low intake of both haem (meat) and nonhaem (vegetarian) sources of iron (e.g., soy) reported in this sample. Highlighting the difficulty in meeting NRV for iron through food intake alone, improved monitoring of iron status is indicated, alongside greater initiatives to improve dietary iron intake among reproductive age Australian women (Barker et al., 2018), particularly among young Australian women, through mandatory food fortification and supplementation (Greig et al., 2013; Leonard et al., 2014). Counseling around optimal dietary sources of micronutrients (e.g., nonhaem iron sources) achieved through subsidized dietetic support for young Australian women, for example, could provider greater opportunity to enhance nutrient intakes through food.

#### 4.2.3 Folate/folic acid

Periconceptional supplementation with 400 μg of folic acid per day is recommended for the primary prevention of NTDs, in addition to adequate dietary folate (Mousa et al., 2019). The rate of periconceptional folic acid supplementation in the current sample (79%–91%) is distinctly higher than rates observed among larger samples of pregnant Australian women (30%–46%) (Conlin et al., 2006; Watson et al., 2006). Given the nature of their involvement in the BABY1000 study, it is likely that many women in this sample were engaged in pregnancy planning and already supplementing with folic acid prior to pregnancy as a result. The true prevalence of folic acid supplementation among the broader population of reproductive age Australian women is likely overrepresented here, especially among those not trying to conceive. In unplanned pregnancies, the protective effects of folic acid supplementation on neural tube closure may be diminished by the time of pregnancy awareness (Berti et al., 2011; Mousa et al., 2019). Wholegrains and fortified breads and cereals are a primary source of dietary folate for Australians, particularly since 2009 when fortification of bread flour with folic acid was first mandated. Prior to this policy, data from the ALSWH revealed that on average, Australian women before and during pregnancy were only meeting approximately half the EAR for folate (~260 mg/day) through food (Hure et al., 2009). While the median reported folate intake of periconceptional women in this study was substantially higher (521 mg/day), a low median ARFS score for grains (6 out of 13 points) indicates further room for improvement.

#### 4.2.4 Iodine

Iodized salt is the primary source of iodine in the Australian diet (Berti et al., 2011), especially since the addition of iodine to breadmaking flour in Australia was mandated in 2009 (Thoma et al., 2011). Interestingly, Charlton et al. found only slight improvements in iodine status among users of iodized salt compared to nonusers during pregnancy (*n* = 139) (Charlton et al., 2010). Iodine is important for fetal brain development, with maternal deficiency predisposing the fetus to intellectual impairment (Mousa et al., 2019). With up to 84% of women in the present sample not meeting the EAR for iodine, public health initiatives should promote the use of iodized salt, alongside other dietary iodine sources including fortified bread, dairy foods, eggs, and appropriate seafood (i.e., low mercury and safely prepared and cooked choices such as salmon and sardines) (Berti et al., 2011).

### 4.3 Strengths and limitations

A key strength of the subset sample of the pilot BABY1000 study includes the collection of dietary data before pregnancy, as it is well‐established that maternal nutrition influences offspring health from the time of conception and throughout pregnancy and breastfeeding (Gluckman et al., 2009; Painter et al., 2008). FFQs have low participant burden, which made it possible to perform multiple measures and capture changes in nutrient intake across pregnancy (Bingham et al., 1994). To avoid overestimating proportions of inadequacy in this sample, micronutrient intake was assessed relative to the EAR, rather than RDI (NHMRC, 2006).

A key limitation was that not all data were complete and available for analysis at the time of data analysis. Measurement error is a limitation inherent to all self‐reported measures of dietary intake (Bingham et al., 1994). In the AES FFQ, foods consumed closest to the time of recall are often overrepresented, while inaccurate estimation of usual frequency and quantity of dietary intake was another probable source of error (Bingham et al., 1994). To improve the validity of the current analyses, AES FFQ responses with biologically implausible total energy intakes defined as <4.5 or >20 MJ/day were excluded (Meltzer et al., 2008).

The current findings may not reflect true “physiological” micronutrient adequacy as the AES FFQ did not adjust for increased dietary requirements for iron and zinc among vegetarian women in light of poor absorption of these nutrients from foods of plant origin (O'Brien et al., 2003). The specific nutrient profiles of dietary supplements consumed could not be accurately calculated and their contribution to usual micronutrient intake was excluded. Although the current results may overestimate the true level of micronutrient inadequacy in this sample, they nonetheless provide a useful benchmark for proportions of food‐derived nutrient inadequacy within the broader population.

Finally, current findings also represent a cohort of women that were more likely to be older, more highly educated and with a lower BMI than the average Australian woman of reproductive age. In 2018, median age of Australian women at parturition was 31 years, compared to 33 years in the present sample (Statistics, 2019). The current sample is also more highly educated than the Australian average, with the observed proportion of women with tertiary level education (96%) exceeding the national proportion (90%) for women aged 20–24 years with Year 12 education or equivalent in 2015 (Statistics, 2016). In 2020, nationally representative data found 47% of pregnant women had a BMI in the “healthy” range, compared to 56% in our cohort (AIHW, 2022).

When these sociodemographic characteristics were considered in relation to diet quality, country of birth was the only variable significantly associated at baseline, and parity in late pregnancy. No other variables were significantly associated with diet quality at either time point. Previous research using the AES has demonstrated several sociodemographic variables are associated with diet quality, including gender, age, socioeconomic status, vegetarian diet, others present at main meals, and country of residence (Whatnall et al., 2022; Williams et al., 2017). It is likely that residual confounders (such as socioeconomic status) and the relatively small sample size of our cohort (especially at 36 weeks) impacted the significance of associations between diet quality and other variables. While country of residence is separate from country of birth, these results and ours highlight how the AES is optimal for use in Australia and those consuming foods most frequently available and consumed by Australians, as the tool is based on Australian dietary guidelines and commonly consumed foods.

### 4.4 Implications for research and practice

Understanding maternal diet quality and patterns in nutrient intake across preconception and pregnancy is crucial to the overall aims of the pilot BABY1000 study. By linking dietary data with prospective health outcomes from this cohort of mother–infant pairs, the current research may help to clarify the relationship between maternal nutrition and offspring health through early life and inform both the larger study and future intervention studies.

Maternal nutrition at the time of conception is also an undeniable determinant of future offspring health (Barker, 1995; Stephenson et al., 2018). Evidence suggests that women are most receptive to dietary change when trying to conceive (M. Barker et al., 2018; Hillier & Olander, 2017). Dietary improvement is often difficult to achieve through pregnancy due to heightened stress and physiological changes, and may be complicated by gastrointestinal symptoms, especially in early pregnancy (Forbes et al., 2018). It follows that prepregnancy nutrition interventions may be a necessary and effective response to optimizing dietary intakes across pregnancy (Barker et al., 2018; Lee, Muggli, et al., 2018; Malek et al., 2016; Mishra et al., 2015). Improvements in prenatal care and public health policy are recommended to optimize maternal diet and support optimal perinatal and long‐term offspring health. Subsidized dietetic treatment for Australian women during pregnancy and ideally, prior to, could enable greater access to personalized, evidence‐based support with the goal of better aligning dietary and supplement intakes with Australian recommendations (Savard et al., 2020).

## 5 CONCLUSIONS

Maternal diet quality and nutrient intakes in the current study sample were largely inconsistent with Australian recommendations and may not be conducive to supporting optimal perinatal or long‐term offspring nutrition‐related health. Key findings include high intakes of saturated fat and suboptimal intakes of iron, iodine, calcium, and folate. Many women may not be aware of the lack of adequacy of their dietary intake or where to find reliable sources of information and support. As healthful dietary habits formed before pregnancy are more likely to extend into future pregnancies, these findings highlight the need for a twofold approach to optimize maternal nutrition before pregnancy. Providing tailored preconception nutrition healthcare to women actively pursuing a pregnancy, as well as nutrition policy initiatives targeting women of childbearing age to help improve maternal diet quality and nutrient intakes, ultimately supporting optimal perinatal and long‐term offspring health. Strong messaging around the importance of prenatal nutrition and the potential consequences of inadequate nutrition during pregnancy and beyond should also be emphasized to help raise awareness and encourage women to seek support and resources and for healthcare providers to make this more available.

## AUTHOR CONTRIBUTIONS

**Kate Maneschi:** Formal analysis (equal); investigation (equal); project administration (equal); writing – original draft (equal). **Taryn Geller:** Formal analysis (equal); investigation (equal); project administration (equal); writing – original draft (equal). **Clare E. Collins:** Writing – review and editing (equal). **Adrienne Gordon:** Conceptualization (equal); funding acquisition (equal); supervision (supporting); writing – review and editing (equal). **Allison Grech:** Conceptualization (equal); formal analysis (equal); methodology (equal); supervision (lead); writing – review and editing (equal).

## FUNDING INFORMATION

This research received no external funding. AG is supported by a Research Training Program (RTP) stipend from the Australian Federal Government.

## CONFLICT OF INTEREST STATEMENT

The authors declare no conflict of interest.

## Supporting information


Tables S1 and S2.

## Data Availability

The data that support the findings of this study are available from the corresponding author upon reasonable request.
