# Supplementary material for: Maternal diet quality and nutrient intakes across preconception and pregnancy are not consistent with Australian guidelines: Results from the pilot BABY1000 study
Source: Food Sci Nutr. 2023 May 4;11(7):4113–23. doi: 10.1002/fsn3.3401 (PMC10345671; doi:10.1002/fsn3.3401)
Supplement: Supplementary file 1 — Tables S1 and S2. [file FSN3-11-4113-s001.docx]

**Supplementary Table 1.** Changes in diet quality and nutrient intake across pregnancy from the pilot BABY1000 study in women with dietary data in preconception/early pregnancy and late pregnancy (*n* = 86).

|  | **Preconception/Early Pregnancy**  (*n* = 86) | | **Late Pregnancy**  (*n* = 86) | | ***p*-value^1^** |
| --- | --- | --- | --- | --- | --- |
|  | **Median**  (IQR) | | **Median**  (IQR) | |  |
| Total energy (kJ) | 8504 (7071–9849) | | 8533 (7447–10221) | | 0.151 |
| Core foods (kJ) | 6160 (5124–7217) | | 6036 (4789–7112) | | 0.589 |
| Core foods (% energy) | 72 (65–78) | | 71 (64–78) | | 0.024 * |
| Non-core foods (kJ) | 2441 (1690–3150) | | 2338 (1768–3423) | | 0.557 |
| Non-core foods (% energy) | 28 (22–35) | | 29 (22–37) | | 0.024 * |
| **ARFS**  (max. points awarded) |  | |  | |  |
| **Total (73)** | 37 (34–43) | | 39 (34–44) | | 0.101 |
| Vegetables (21) | 15 (11–17) | | 14 (11–16) | | 0.801 |
| Grains (13) | 6 (5–7) | | 6 (5–7) | | 0.380 |
| Fruit (12) | 7 (5–8) | | 7 (5–8) | | 0.675 |
| Dairy (11) | 4 (3–5) | | 5 (3–6) | | 0.002 ** |
| Meat (7) | 3 (2–4) | | 3 (2–4) | | 0.086 |
| Meat alternatives (6) | 3 (2–4) | | 3 (2–4) | | 0.504 |
| Extras (2) | 1 (0–1) | | 1 (0–1) | | 0.400 |
| Water (1) | 1 (1–1) | | 1 (1–1) | | 0.020 * |
| **Nutrient** | **Median**  (IQR) | **Meeting**  *n* (%) | **Median**  (IQR) | **Meeting**  *n* (%) |  |
| Vitamin B_12_ (µg) | 3.4 (2.6–4.3) | 67 (78) | 3.6 (3.0–4.4) | 76 (88) | 0.066 |
| Calcium (mg) | 839 (579–1043) | 20 (23) | 840 (691–1097) | 27 (31) | 0.051 |
| Folate (µg) ‡ | 530 (402–655) | 45 (52) | 538 (409–658) | 46 (53) | 0.613 |
| Iodine (µg) | 119 (88–150) | 13 (15) | 134 (99–159) | 22 (26) | 0.002 ** |
| Iron (mg) | 10 (8–13) | 0 (0) | 11 (9–13) | 0 (0) | 0.296 |
| Zinc (mg) | 11 (9–13) | 65 (76) | 11 (10–13) | 73 (85) | 0.169 |
|  | **Median** †  (IQR) | | **Median** †  (IQR) | | ***p*-value^1^** |
| Protein | 17 (16–20) | | 18 (16–19) | | 0.478 |
| Carbohydrate | 45 (41–49) | | 45 (41–49) | | 0.899 |
| Total fat | 36 (33–39) | | 37 (34–40) | | 0.054 |
| Saturated fat | 13 (12–15) | | 14 (12–16) | | 0.006 ** |

ARFS, Australian Recommended Food Score; AMDR, Acceptable Macronutrient Distribution Range; IQR, Interquartile Range; †, Macronutrient intake expressed as a percentage of total energy intake; ‡, Dietary folate equivalents; –, Percent is not calculated for missing values; ^1^ Wilcoxon signed-ranks test was performed to identify statistically significant differences between median values. Significance level; *, Significant (*p* ≤ 0.05); **, Very significant (*p* ≤ 0.01).

**Supplementary Table 2.** Examining micronutrient intake relative to the ARFS in women from the pilot BABY1000 study at late pregnancy (*n* = 99). Women were stratified into tertiles based on total ARFS and a comparison was drawn between those with the lowest ARFS (Tertile 1) (*n* = 37) and the highest ARFS (tertile 3) (*n* = 32).

|  | **Tertile 1 (ARFS <36)**  (*n* = 37) | **Tertile 2 (ARFS 37–41)**  (*n* = 30) | **Tertile 3 (ARFS >42)**  (*n* = 32) | ***p*-value^1^** |
| --- | --- | --- | --- | --- |
|  | **Median**  (IQR) | **Median**  (IQR) | **Median**  (IQR) |  |
| ARFS | 33 (29–35) | 39 (38–40) | 46 (44–48) |  |
| **Micronutrient** |  |  |  |  |
| Vitamin B_12_ (µg) | 3.2 (2.3–4.4) | 3.6 (3.1–4.2) | 3.9 (3.3–5.3) | 0.020 * |
| Calcium (mg) | 697 (631–928) | 926 (772–1117) | 963 (823–1167) | 0.006 ** |
| Folate (µg) ‡ | 481 (359–587) | 557 (464–693) | 562 (505–689) | 0.002 ** |
| Iodine (µg) | 102 (82–133) | 141 (117–159) | 144 (117–180) | 0.001 ** |
| Iron (mg) | 8.8 (7.6-11) | 12 (10–14) | 12 (10-13) | 0.001 ** |
| Zinc (mg) | 10 (8.4–11) | 12 (10–14) | 12 (10-14) | 0.067 |

ARFS, Australian Recommended Food Score; IQR, Interquartile Range; ‡, Dietary folate equivalents; ^1^ Two-sample t-Test assuming unequal variances was performed to identify statistically significant differences between tertile 1 (ARFS <36) and tertile 3 (ARFS >42). Significance level; *, Significant (*p* ≤ 0.05); **, Very significant (*p* ≤ 0.01).
